# Nomograms to predict lung metastasis in malignant primary osseous spinal neoplasms and cancer-specific survival in lung metastasis subgroup

**DOI:** 10.3389/fonc.2024.1393990

**Published:** 2024-08-20

**Authors:** Yong Jiang, Yapeng Zhu, Yongli Ding, Xinchang Lu

**Affiliations:** ^1^ Orthopaedic Department, The First Affiliated Hospital of Henan University of Chinese Medicine, Zhengzhou, Henan, China; ^2^ Department of Orthopaedic Surgery, The First Affiliated Hospital of Zhengzhou University, Zhengzhou, Henan, China

**Keywords:** spinal tumors, SEER database, lung metastasis, survival analysis, nomogram

## Abstract

**Purpose:**

To construct and validate nomograms for predicting lung metastasis probability in patients with malignant primary osseous spinal neoplasms (MPOSN) at initial diagnosis and predicting cancer-specific survival (CSS) in the lung metastasis subgroup.

**Methods:**

A total of 1,298 patients with spinal primary osteosarcoma, chondrosarcoma, Ewing sarcoma, and chordoma were retrospectively collected. Least absolute shrinkage and selection operator (LASSO) and multivariate logistic analysis were used to identify the predictors for lung metastasis. LASSO and multivariate Cox analysis were used to identify the prognostic factors for 3- and 5-year CSS in the lung metastasis subgroup. Receiver operating characteristic (ROC) curves, calibration curves, and decision curve analyses (DCA) were used to estimate the accuracy and net benefits of nomograms.

**Results:**

Histologic type, grade, lymph node involvement, tumor size, tumor extension, and other site metastasis were identified as predictors for lung metastasis. The area under the curve (AUC) for the training and validating cohorts were 0.825 and 0.827, respectively. Age, histologic type, surgery at primary site, and grade were identified as the prognostic factors for the CSS. The AUC for the 3- and 5-year CSS were 0.790 and 0.740, respectively. Calibration curves revealed good agreements, and the Hosmer and Lemeshow test identified the models to be well fitted. DCA curves demonstrated that nomograms were clinically useful.

**Conclusion:**

The nomograms constructed and validated by us could provide clinicians with a rapid and user-friendly tool to predict lung metastasis probability in patients with MPOSN at initial diagnosis and make a personalized CSS evaluation for the lung metastasis subgroup.

## 1 Introduction

Malignant primary osseous spinal neoplasms (MPOSN) are very rare and make up less than 5% of all bone tumors (1). MPOSN mainly consist of osteosarcoma (35.1%), chondrosarcoma (25.8%), Ewing sarcoma (16.0%), and chordoma (8.4%) (2–4). Malignant primary osseous neoplasms occur primarily in extremities and rarely in the spine (5–7). However, when it occurs in the spine, the prognosis is poor. Extensive excision is effective and recommended for primary malignant bone tumors in the extremities, but extensive excision is more difficult and challenging in the spine, and is often dangerous (8). Moreover, previous studies have reported higher rates of lung metastasis of malignant tumors in the spine compared to other sites such as the extremities (9, 10). Zhang et al. suggested that occurring at the axial site, such as the spine, was significantly related to lung metastasis of malignant bone tumors such as osteosarcoma and chondrosarcoma (9). Although medical technology has developed increasingly in recent years, the survival rate of the patients with distant metastasis still remained poor (11, 12). It was worth noticing that the lungs have been proven as the most metastasized site, and the incidence of lung metastasis at initial diagnosis was approximately 10%–40% in malignant primary osseous neoplasms (13–16). Previous studies have demonstrated that the patients with MPOSN benefited greatly from the early diagnosis of lung metastasis (17–19). Considering the micro-metastases in malignant tumors and the moderate performance of conventional lung CT scan in the detection of small lung nodules (20, 21), combining clinicopathologic characteristics with imaging features may help to improve the accuracy of initial diagnosis. For those with MPOSN who have already presented lung metastasis, the early identification of survival rate can also help provide individual adjuvant therapies or trial options.

Previous literatures have reported many potential predictors for lung metastasis or survival rate in spine tumors (22, 23). However, the role of only a single variable may be limited. A predictive tool, which can integrate multiple significant risk features to make an individual prediction is urgently needed. The nomogram has been confirmed to provide a superior individual disease risk estimation and promote the decision management of treatment (22). To our limited knowledge, a nomogram for predicting cancer-specific survival (CSS) of lung metastasis subgroup in patients with MPOSN has not yet been reported. Moreover, the nomogram to predict lung metastasis probability in MPOSN at initial diagnosis was also rare and needs further large-sample investigation (10).

In the current study, the corresponding data from the Surveillance, Epidemiology, and End Results (SEER) database, which originates from 17 geographically variable cancer registries and represents approximately 26% of the US population were collected (24). The purpose of this study was to construct and validate nomograms for predicting lung metastasis probability in patients with MPOSN at initial diagnosis and predicting CSS in the lung metastasis subgroup.

## 2 Materials and methods

### 2.1 Patient cohort

The inclusion criteria were as follows: (1) diagnosed with the most common types of MPOSN (osteosarcoma, chondrosarcoma, Ewing sarcoma, and chordoma) in the SEER database between 2004 to 2015; (2) the primary sites of tumors were the spine; (3) microscopically confirmed, positive histology confirmed, or positive exfoliative cytology confirmed; and (3) known survival months and status.

The exclusion criteria were as follows: (1) unknown metastasis status; (2) unknown race; (3) unknown tumor size and unknown tumor extension; and (3) unknown surgery in primary site, unknown radiotherapy, and unknown lymph nodes removed.

Clinicopathologic features were as follows: (1) demographics (age, race, sex, year of birth, reporting source, insurance, and marriage); (2) tumor characteristics (tumor size, tumor extension, histologic type, grade, original laterality, lymph node involvement, and metastasis status); and (3) therapies (surgery at primary site, lymph nodes removed, radiotherapy, and chemotherapy) and survival data were identified.

### 2.2 Statistical analysis

The collected data (n = 1,298) were randomly assigned into a training cohort (n = 910) and a validation cohort (n = 388). The baseline clinicopathologic features between the two groups were compared via Chi-square test. Least absolute shrinkage and selection operator (LASSO) regression was performed to initially select the most significant predictive features and ensure that the multiple factor models were not over fitting. Multivariate logistic regression was used to identify the ultimate independent risk factors for lung metastasis prediction. In the lung metastasis subgroup, the ultimate prognostic factors for CSS were identified by multivariate Cox regression.

Based on the ultimate selected variables, the nomogram for lung metastasis prediction was constructed and internally validated in the training cohort and externally validated in the validation cohort. In the lung metastasis subgroup, the nomogram for the CSS was constructed and internally validated in the cancer-specific cases. The evaluation for the predictive discrimination of nomograms were performed via receiver operating characteristic (ROC) curves and the areas under the curves (AUC). Decision curve analyses (DCAs) were utilized to assess the clinical usefulness and net benefits of the prediction models (25, 26). Meanwhile, calibration curves and Hosmer–Lemeshow tests were used to validate the concordance of nomograms. Kaplan–Meier curves were plotted to perform to construct cumulative survival curves. The statistical significance was evaluated by Log-rank test.

All of these statistical analyses and graphics were performed by SPSS statistics software version 22.0 (IBM Corporation, Armonk, NY, USA), R software (3.6.3), and Rstudio software (1.2.5033). Two-sided p value <0.05 was defined to have statistical significance.

## 3 Results

### 3.1 Patient baseline characteristics

A total of 2,168 patients based on the inclusion criteria were selected from the SEER database during the period of 2004–2015. In total, 870 patients were excluded according to the exclusion criteria. Ultimately, 1,298 cases were determined and randomly divided into the training cohort (n = 980) and the validation cohort (n = 338). There were no significant differences between the two cohorts (**Table 1**, p > 0.05). In the lung metastasis subgroup, 4 cases who died due to causes other than cancer were excluded, and 122 cases were determined ultimately.

**Table 1:** Distribution of demographic and clinical information.

Variables	Total population (N = 1,298; 100.0%)		Training cohort (N = 910; 70.1%)		Validation cohort (N = 388; 29.9%)		P
	N	%	N	%	N	%	
**Lung metastasis**							0.945
No	1,172	90.3	822	90.3	350	90.2	
Yes	126	9.7	88	9.7	38	9.8	
**Age (years)**							0.432
<18	197	15.2	136	14.9	61	15.7	
18–50	476	36.7	344	37.8	132	34.0	
>50	625	48.2	430	47.3	195	50.3	
**Race**							0.115
White	1,110	85.5	789	86.7	321	82.7	
Black	90	6.9	55	6.0	35	9.0	
Other	98	7.6	66	7.3	32	8.2	
**Sex**							0.086
Male	779	60.0	560	61.5	219	56.4	
Female	519	40.0	350	38.5	169	43.6	
**Year of birth**							0.965
<1970	779	60.0	548	60.2	231	59.5	
1970–2000	474	36.5	331	36.4	143	36.9	
>2000	45	3.5	31	3.4	14	3.6	
**ICD O 3 histologic type**							0.233
Osteosarcoma	239	18.4	161	17.7	78	20.1	
Chondrosarcoma	433	33.4	301	33.1	132	34.0	
Ewing’s sarcoma	280	21.6	191	21.0	89	22.9	
Chordoma	346	26.7	257	28.2	89	22.9	
**Grade**							0.338
Grade I	147	11.3	98	10.8	49	12.6	
Grade II	188	14.5	131	14.4	57	14.7	
Grade III	138	10.6	90	9.9	48	12.4	
Grade IV	197	15.2	135	14.8	62	16.0	
Unknown	628	48.4	456	50.1	172	44.3	
**Lymph node involvement**							0.245
No	1,198	92.3	845	92.9	353	91.0	
Yes	100	7.7	65	7.1	35	9.0	
**Other site metastasis**							0.830
No	1,147	88.4	803	88.2	344	88.7	
Yes	151	11.6	107	11.8	44	11.3	
**Tumor size (cm)**							0.151
<5	267	20.6	189	20.8	78	20.1	
5–10	545	42.0	367	40.3	178	45.9	
>10	486	37.4	354	38.9	132	34.0	
**Tumor extension**							0.402
Inside periosteum	457	35.2	327	35.9	130	33.5	
Beyond periosteum	841	64.8	583	64.1	258	66.5	
**Origin laterality**							0.471
Left	292	22.5	204	22.4	88	22.7	
Right	321	24.7	217	23.8	104	26.8	
Other	685	52.8	489	53.7	196	50.5	
**Reporting source**							0.688
Hospital	1,275	98.2	893	98.1	382	98.5	
Other	23	1.8	17	1.9	6	1.5	
**Insurance**							0.961
Insured	843	64.9	591	64.9	252	64.9	
Medicaid	165	12.7	117	12.9	48	12.4	
Other	290	22.3	202	22.2	88	22.7	
**Marriage**							0.839
Married	585	45.1	417	45.8	168	43.3	
Divorced	67	5.2	49	5.4	18	4.6	
Single	534	41.1	366	40.2	168	43.3	
Widowed	62	4.8	44	4.8	18	4.6	
Other	50	3.9	34	3.7	16	4.1	

Chi-square test: these values are statistically significant at a p-value <0.05.

### 3.2 Prognostic factor selection

**Figure 1:**
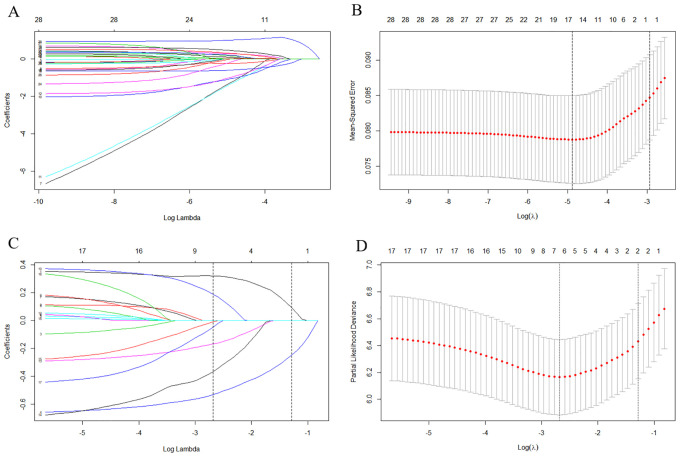
The results of the least absolute shrinkage and selection operator (LASSO) regression. The LASSO regression analysis initially selected age, race, year of birth, grade, histology type, origin laterality, tumor size, tumor extension, lymph nodes involvement, other site metastasis, insurance and marriage as the risk factors for lung metastasis **(A, B)**, and age, histology type, other metastasis, grade, surgery at primary site, lymph nodes removed and chemotherapy as prognostic factors for the prediction of OS in the lung metastasis subgroup **(C, D)**. LASSO, Least absolute shrinkage and selection operator; OS, Overall survival.

Because the exact time of therapies were unknown, we first excluded surgery at the primary site, lymph node removal, radiotherapy, and chemotherapy from the prediction for lung metastasis. LASSO regression analysis initially selected the significant factors, including age, race, year of birth, grade, histologic type, original laterality, tumor size, tumor extension, lymph node involvement, other site metastasis, insurance, and marriage (**Figures 1A, B**). The variables, including grade, histologic type, tumor size, tumor extension, lymph node involvement, and other site metastasis were identified as the ultimate risk factors for lung metastasis via multivariate logistic regression (**Table 2**, p < 0.05).

**Table 2:** Multivariate logistic regression for analyzing the lung metastasis-associated factors in the training cohort (n = 910).

Variables	Training cohort (N = 910)	
	OR (95% CI)	P
Age(years)		
<18	1 (reference)	
18–50	0.936 (0.358–2.547)	0.894
>50	1.010 (0.562–1.826)	0.972
Race		
White	1 (reference)	
Black	0.678 (0.233–1.872)	0.460
Other	0.783 (0.397–1.626)	0.495
Year of birth		
<1970	1 (reference)	
1970–2000	0.516 (0.126–2.005)	0.346
>2000	0.754 (0.322–1.849)	0.526
Grade		
Grade I	1 (reference)	
Grade II	0.416 (0.021–2.892)	0.438
Grade III	4.231 (1.422–15.664)	0.016*
Grade IV	5.151 (1.732–19.170)	0.006*
Unknown	4.599 (1.583–16.839)	0.010*
ICD O 3 Histologic type		
Osteosarcoma	1 (reference)	
Chondrosarcoma	1.647 (0.748–3.716)	0.221
Ewing Sarcoma	0.222 (0.067–0.639)	0.008*
Chordoma	1.587 (0.831–3.111)	0.168
Origin laterality		
Left	1 (reference)	
Right	1.346 (0.816–2.235)	0.246
Other	0.667 (0.390–1.139)	0.138
Tumor size (cm)		
<5.0	1 (reference)	
5.0–10.0	1.447 (0.680–3.377)	0.361
>10.0	2.182 (1.045–5.022)	0.049*
Tumor extension		
Inside periosteum	1 (reference)	
Beyond periosteum	0.445 (0.244–0.771)	0.006*
Lymph node involvement		
No	1 (reference)	
Yes	1.869 (1.046–3.264)	0.031*
Other site metastasis		
No	1 (reference)	
Yes	2.316 (1.430–3.729)	0.001*
Insurance		
Insured	1 (reference)	
Medicaid	1.479 (0.841–2.557)	0.166
Other	0.722 (0.415–1.218)	0.233
Marriage		
Married	1 (reference)	
Divorced	2.373 (0.628–15.797)	0.988
Single	2.845 (0.698–19.702)	0.199
Widowed	1.462 (0.206–12.783)	0.705
Other	3.294 (0.520–27.928)	0.220

Multivariable logistic regression: (*) at a p-value <0.05.

Grade I, well differentiated; Grade II, moderately differentiated; Grade III, poorly differentiated; Grade IV, undifferentiated.

Moreover, LASSO regression analysis initially selected variables, including age, histologic type, grade, other site metastasis, surgery at primary site, lymph node removed, and chemotherapy (**Figures 1C, D**) in the lung metastasis subgroup. Further, the variables, including age, histologic type, grade, and surgery at the primary site, were identified as the ultimate prognostic factors for the CSS via multivariate Cox regression analysis (**Table 3**, p < 0.05).

**Table 3:** Multivariate Cox regression for analyzing the prognosis-associated factors in the metastasis subgroup (n = 122).

Variables	Multivariate analysis	
	Hazard ratio (95% CI)	p-Value
Age (years)		
<18	1 (reference)	
18–50	1.438 (0.757–2.732)	0.267
>50	2.705 (1.237–5.916)	0.013*
ICD O 3 histologic type		
Osteosarcoma	1 (reference)	
Chondrosarcoma	0.965 (0.476–1.955)	0.000*
Ewing’s sarcoma	0.259 (0.135–0.494)	0.007*
Chordoma	0.100 (0.019–0.536)	0.922
Other site metastasis		
No	1 (reference)	
Yes	1.464 (0.918–2.335)	0.109
Grade		
Grade I	1 (reference)	
Grade II	0.869 (0.103–7.333)	0.897
Grade III	0.267 (0.072–0.988)	0.048*
Grade IV	0.923 (0.460–1.851)	0.821
Unknown	1.165 (0.680–1.995)	0.579
Surg prim site		
No	1 (reference)	
Yes	0.544 (0.301–0.984)	0.044*
Lymph nodes removed		
No	1 (reference)	
Yes	0.900 (0.309–2.622)	0.846
Chemotherapy		
No/unknown	1 (reference)	
Yes	0.702 (0.363–1.358)	0.293

Multivariable Cox regression: (*) at a p-value <0.05.

Grade I, well differentiated; Grade II, moderately differentiated; Grade III, poorly differentiated; Grade IV, undifferentiated.

### 3.3 Construction and validation of the nomogram

**Figure 2:**
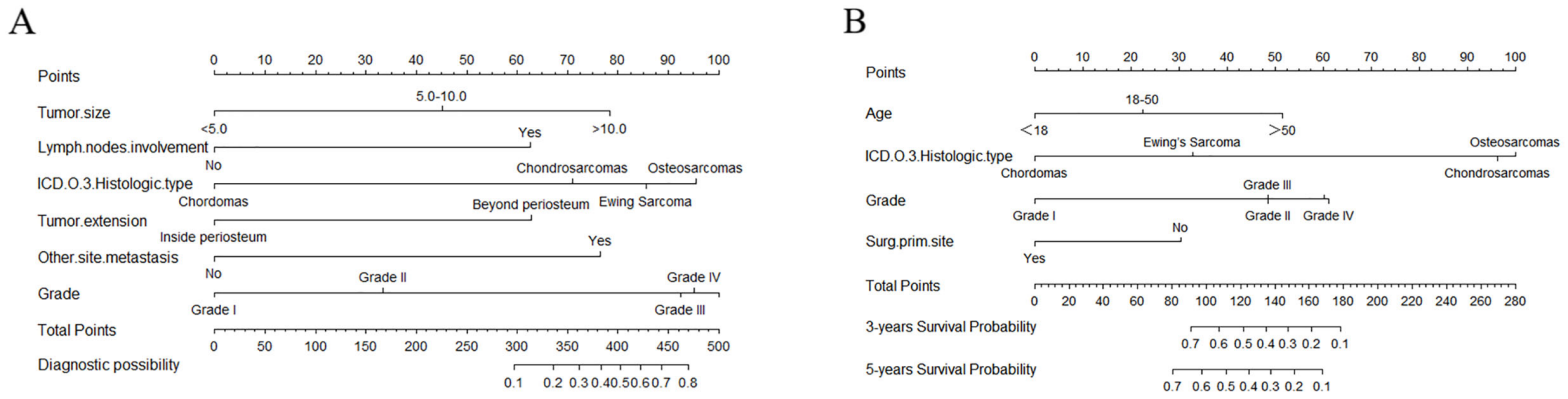
Nomograms for predicting the probability of lung metastasis **(A)** in patients with malignant primary osseous spinal neoplasms (MPOSN), and the 3- and 5-year cancer-specific survival (CSS) in the lung metastasis subgroups **(B)** (Grade I, well differentiated; Grade II, moderately differentiated; Grade III, poorly differentiated; Grade IV, undifferentiated).

**Figure 3:**
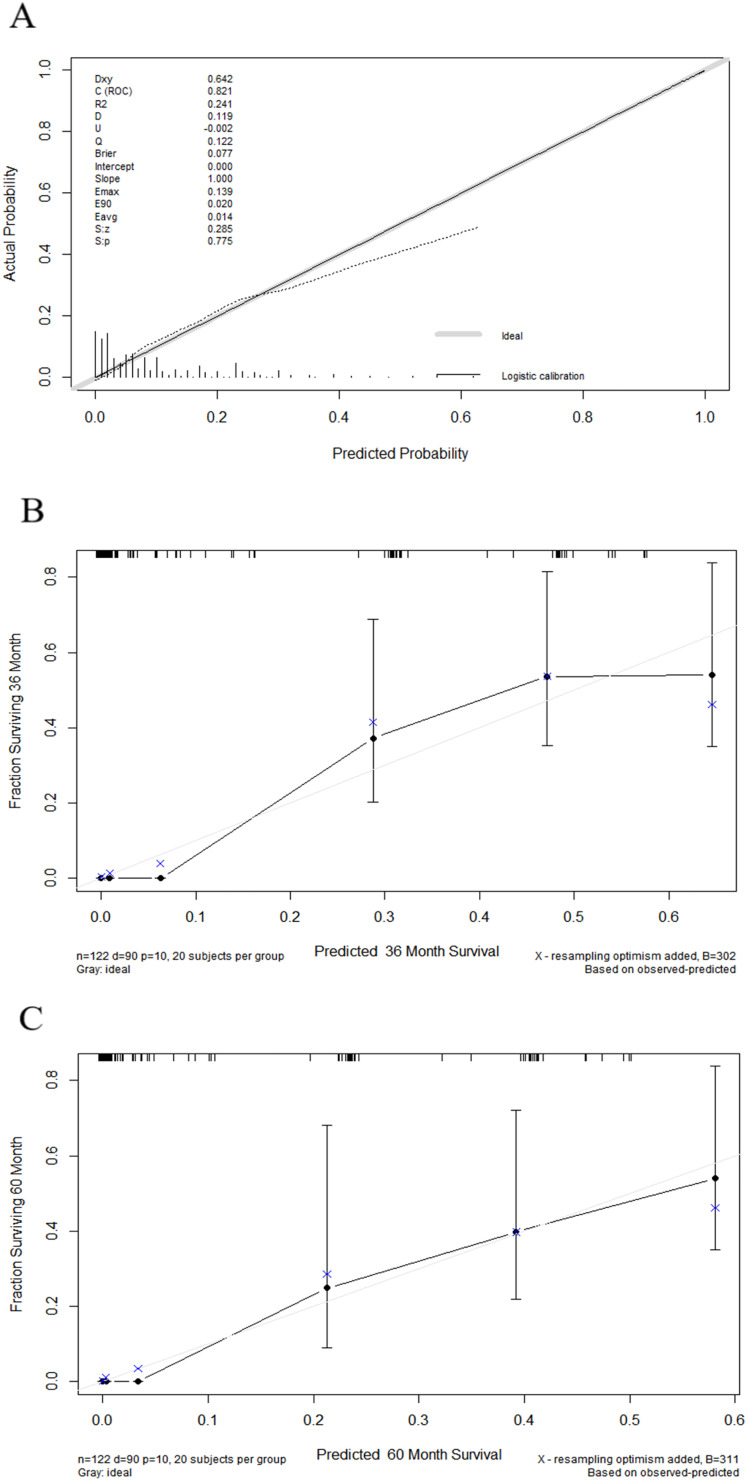
Calibration curves showed the presentable accuracy of nomograms by comparing nomogram predictions with actual endpoints. (1) Calibration curve for nomogram A **(A)**. (2) Calibration curves for nomogram B **(B, C)**.

**Figure 4:**
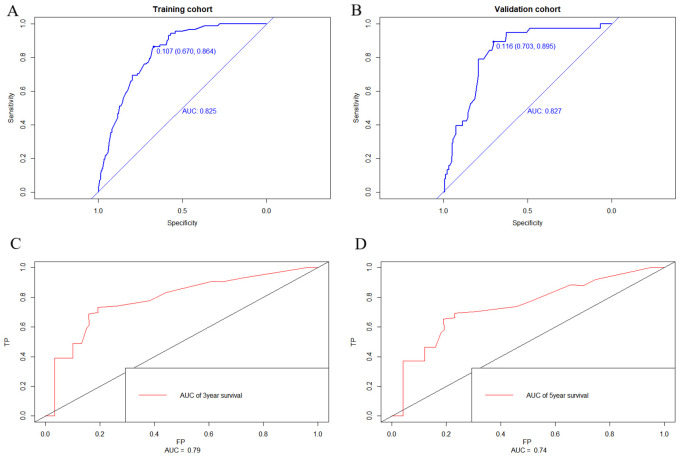
Receiver operating characteristic (ROC) curve of the nomograms. The areas under the curves (AUC) of training and validating cohorts in nomogram A were 0.825 and 0.827, respectively **(A, B)**. The AUC of 3- and 5-year CCS in nomogram B were 0.790 and 0.740, respectively **(C, D)** appearing as good predictive discrimination.

Nomograms were constructed as follows to predict the probability of lung metastasis (**Figure 2A**, nomogram A) and the 3- and 5-year CSS in the lung metastasis subgroup (**Figure 2B**, nomogram B). The calibration curves for the two nomograms approached the ideal match straight line indicating that they are well calibrated (**Figures 3A–C**). The Hosmer and Lemeshow test identified the models as well fitted. The AUC of training and validating cohorts in the nomogram A were 0.825 and 0.827, respectively (**Figures 4A, B**). The AUC of 3- and 5-year CCS in the nomogram B were 0.790 and 0.740, respectively (**Figures 4C, D**), appearing with good predictive discrimination.

**Figure 5:**
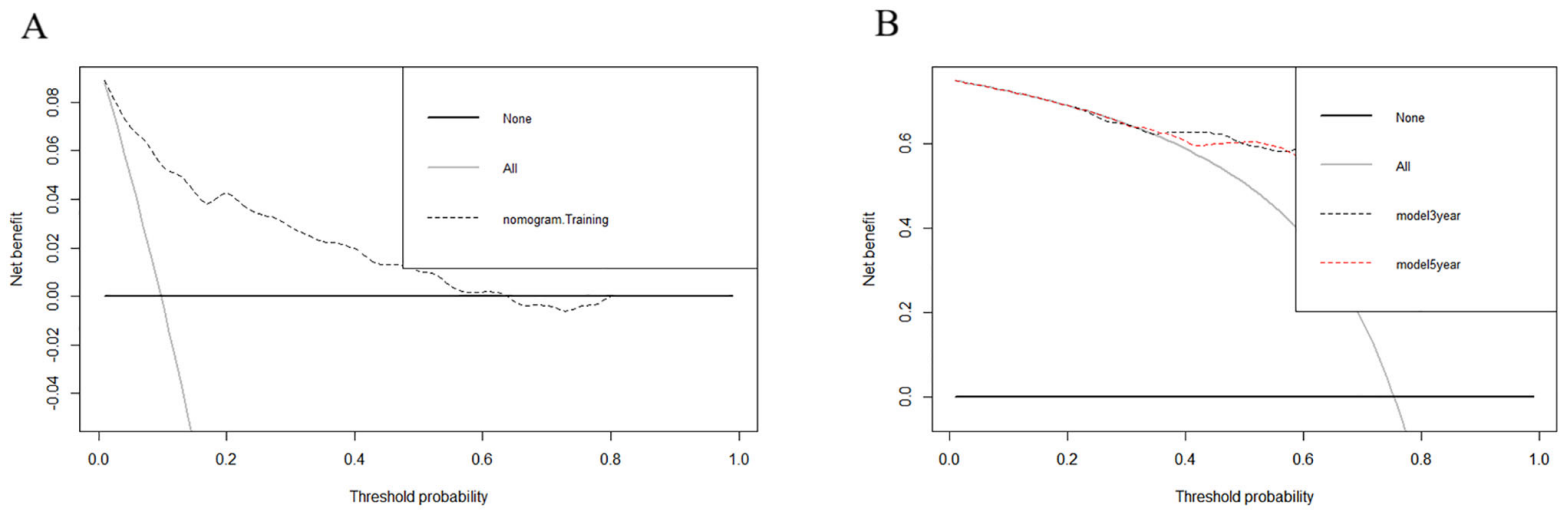
Decision curve analyses (DCA) showed that the nomograms provided clinical usefulness and net benefits. (1) DCA for nomogram A **(A)**. (2) DCA for nomogram B **(B)**.

**Figure 6:**
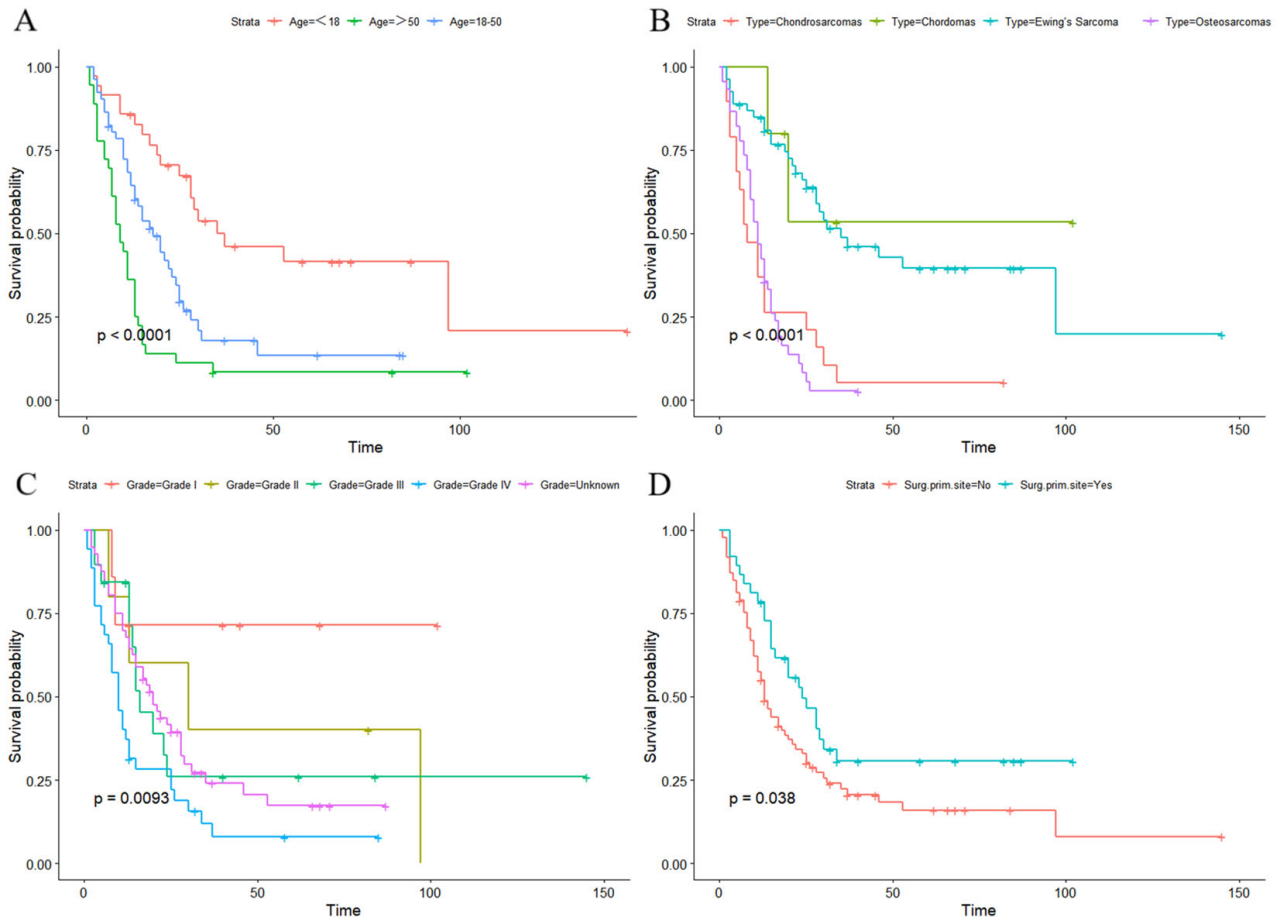
Kaplan–Meier curves and log-rank analyses demonstrated that advanced age **(A)**, histologic type **(B)**, high tumor grade **(C)**, and those who did not undergo surgery at primary site **(D)** were associated with worse prognoses in the lung metastasis subgroup.

DCA showed that the nomograms provided clinical usefulness and net benefits (**Figures 5A, B**). Kaplan–Meier curves and log-rank analyses demonstrated that advanced age (p < 0.001, **Figure 6A**), histologic type (p < 0.001, **Figure 6B**), high tumor grade (p = 0.009, **Figure 6C**), and those who did not undergo surgery at the primary site (p < 0.038, **Figure 6D**) were associated with worse prognoses.

## 4 Discussion

In recent years, nomograms have generally been used as a predictive tool for individual diagnosis or survival outcome (27, 28). For patients with MPOSN, nomograms can meet our desire for improving the diagnostic rate of lung metastasis and identifying the high-risk patients in the lung metastasis subgroup at an early stage.

In the present study, six characteristics were identified as the independent risk factors for lung metastasis, including grade, histologic type, other site metastasis, tumor size, tumor extension, and lymph node involvement. Similar results have been shown in the previous literatures. Xie et al. (22) investigated data of 4,459 patients with malignant primary osseous neoplasms and found variables, including histology type (osteosarcoma and Ewing sarcoma), lager tumors, or higher tumor grade were associated with higher possibility of lung metastasis. Fan et al. (10) reported that higher American Joint Committee on Cancer (AJCC) T stage, higher AJCC N stage, and tumor extension beyond the periosteum independently contributed to lung metastasis in MPOSN. Besides, LASSO regression and multivariate logistic regression analyses in our study demonstrated that other site metastasis was a novel risk factor to predict lung metastasis. The reason may be that the lungs are the most common site of metastasis in MPOSN, so once distant metastases occurred, whether it was detected or not, the occurrence of metastasis to the lungs was theoretically high.

In the lung metastasis subgroup, our survival analysis revealed that advanced age was related to poorer prognosis. A possible explanation is that older patients tended to have higher pathological grades and larger tumor sizes, which have been reported to correlate with survival of bone tumors (29). In our result, the possibility of histologic type for predicting lung metastasis from high to low were, respectively, osteosarcoma, chondrosarcoma, Ewing sarcoma, and chordoma. Previous studies have showed that 5-year survival rates were approximately 20%–30% in osteosarcoma patients with lung metastasis (30), and the rates were respectively 50% in Ewing sarcoma (11) and 45.7% in chondrosarcoma (31). Compared to the above, chordoma is a type of relatively slow-growing and low-grade malignancy, the 5-year overall survival was between 50% and 75%, and distant metastasis is rare (32, 33).

Mukherjee et al. (12) reported that independent of other factors, patients undergoing surgical resection of primary spinal chordoma, chondrosarcoma, Ewing sarcoma, or osteosarcoma all showed prolonged survival. They also found an interesting result that adjuvant radiotherapy can improve the survival only in patients with osteosarcoma and chordomas who underwent surgical resection. Similarly, radiotherapy and chemotherapy were not identified as independent prognostic factors in our study. This phenomenon may be due to some subclassifications of radiotherapy and chemotherapy in the SEER database, which were unknown due to the unavailable information. Meanwhile, different tumors have different responses to radiotherapy and chemotherapy (34, 35). Last but not the least, the effects of adjuvant radiotherapy and chemotherapy may be associated with surgical resection to some extent (36–41).

Based on the identified variables, we constructed and validated nomograms A and B (**Figures 2A, B**). For instance, a patient was diagnosed as having chondrosarcoma with beyond periosteum tumor extension and liver metastasis, and he did not undergo surgery at the primary site, and his tumor grade was III (poorly differentiated). Besides, the tumor size was 12 cm, and he had lymph node involvement. To use nomogram A (**Figure 2A**), we draw a perpendicular line from each predictive factor to obtain the corresponding points. By adding up each point, he gets approximately 435 total points, and we rapidly conclude his lung metastasis probability is approximately 65%. Based on the result, if the conventional lung CT scan revealed nothing, we may suggest the patient take further detection such as high-resolution CT or PET-CT.

There are also some limitations in the present study. First, this is a retrospective study, which may contain a latent risk of bias. Second, the internal and external validations of nomogram A were based on the same center. It may be more reliable to validate nomograms in different centers. Third, due to fewer sample data, we have to analyze the four malignancies together. We expect that more cases would be included in further prospective studies, and the cancer-specific survival analysis of lung metastasis would be carried out separately for four tumor types. Moreover, we did not have external validation of nomogram B. Last but not the least, because the factors, such as pathologic fracture, genetic, and epigenetic factors, were not found in the SEER database, they were not included in the study.

## 5 Conclusion

The nomograms constructed and validated by us could provide clinicians with a rapid and user-friendly tool to predict lung metastasis probability in patients with MPOSN at initial diagnosis and make a personalized CCS evaluation for the lung metastasis subgroup.

## Data Availability

The original contributions presented in the study are included in the article/supplementary material. Further inquiries can be directed to the corresponding authors.
